# Marginal ice zone dynamics: future research perspectives and pathways

**DOI:** 10.1098/rsta.2021.0267

**Published:** 2022-10-31

**Authors:** L. G. Bennetts, C. M. Bitz, D. L. Feltham, A. L. Kohout, M. H. Meylan

**Affiliations:** ^1^ School of Mathematical Sciences, University of Adelaide, Adelaide, South Australia 5005, Australia; ^2^ Department of Atmospheric Sciences, University of Washington, Seattle, WA, USA; ^3^ Department of Meteorology, University of Reading, Reading, UK; ^4^ National Institute of Water and Atmospheric Research, Christchurch, New Zealand; ^5^ School of Mathematical and Physical Sciences, University of Newcastle, Callaghan, New South Wales 2308, Australia

**Keywords:** marginal ice zone, sea ice, climate change

## Abstract

Perspectives are discussed on future directions for the field of marginal ice zone (MIZ) dynamics, based on the extraordinary progress made over the past decade in its theory, modelling and observations. Research themes are proposed that would shift the field’s focus towards the broader implications of MIZ dynamics in the climate system. In particular, pathways are recommended for research that highlights the impacts of trends in the MIZ on the responses of Arctic and Antarctic sea ice to climate change.

This article is part of the theme issue ‘Theory, modelling and observations of marginal ice zone dynamics: multidisciplinary perspectives and outlooks’.

## 1. Introduction

The prevailing phase of growth in marginal ice zone (MIZ) dynamics research can be traced back to a group of seminal studies published approximately a decade ago. A notable example is the wave–ice interaction model (WIM) proposed by Dumont *et al.* [1] and Williams *et al.* [2,3], in which theories of wave attenuation in the MIZ and wave-induced floe breakup are coupled in a form that has allowed for implementation in numerical wave and sea ice models. The study by Doble & Bidlot [4] has also been influential. It contains a comparison of wave activity in the MIZ predicted by a numerical wave model against long-term observations (over one season) from a drifting wave buoy, where the observations leverage on technological advances in satellite links and low-power microprocessors. Reductions in Arctic sea ice extent set the backdrop for the studies, with [1–3] emanating from the *Waves in Ice Forecasting for Arctic Operators* project that aimed to improve safety forecasts for human presence in the emerging Arctic Ocean, and [4] (despite presenting Antarctic observations) motivated by the role of wave-induced ice breakup in the dramatic 2007 Arctic summer sea ice loss event [5].

The success of the studies by [1–4] can largely be attributed to the different path down which they took the field of MIZ dynamics. The authors expressed the view that the theory of wave–ice interactions had matured to a stage at which the focus should shift to incorporating simplified forms of the theories in numerical models for application to real-world situations, and were motivated by the theories for wave attenuation in the MIZ published around that time [6–8]. Multiple research groups have taken up the challenge of embedding MIZ dynamics into wave and sea ice models, and the latest advances are reported in this theme issue [9–11]. Observations of MIZ dynamics have increased in concert, including wave attenuation, floe size distributions (FSDs) and ice dynamics. In turn, findings derived from model and observational data have motivated reassessments of existing theories, e.g. of wave attenuation [12], new theories, e.g. of FSD emergence and evolution [13], and laboratory experimental models, e.g. of wave attenuation and ice breakup [14].

Therefore, greater interplay between the theory, modelling and observational communities has been crucial in driving contemporary MIZ dynamics research, and the theme issue aims to accelerate advances by bringing the communities even closer together. The theme issue also looks towards a future of ongoing growth of MIZ dynamics research. The purpose of this closing article is to collect together the future perspectives outlined by the authors of the three mini-reviews and delineate future pathways that will generate broader impact of MIZ dynamics research.

## 2. Future perspectives from the mini-reviews

The MIZ is characterized by interactions between the ice cover and ocean waves. The topic of wave–ice interactions underpins MIZ dynamics and has dominated the research focus to date. The three mini-reviews [15–17] outline how the focus should evolve towards studying the role of wave–ice interactions in the atmosphere–ice–ocean–land feedback system, which will better constrain the aspects of wave–ice interactions that should be prioritized for greater attention.

Horvat [16] and Dumont [17] take perspectives on improved understanding of sea ice trends and projecting future scenarios. Horvat [16] picks up on the potential wave–ice feedbacks that have strongly motivated MIZ dynamics research over the past decade. There is a positive (summer) feedback, which involves a wave-induced ice breakup event weakening the ice cover, hence allowing energetic waves to propagate deeper into the ice-covered ocean, which further weakens the ice cover, and so on. There is also a negative (winter) feedback in which increased wave activity in the MIZ strengthens the ice cover, e.g. by promoting ice growth, thus reducing wave activity in the MIZ, which allows the ice cover to strengthen, and so on. Horvat notes only a handful of studies have implicated the positive feedback in relationships between sea ice evolution and wave activity, and no existing studies analyse the negative feedback. He points to the possibility of using laboratory experiments to analyse the feedbacks under controlled conditions.

Dumont [17] discusses important unknowns in MIZ ice mass and momentum balances. He identifies prominent gaps in our current understanding of the MIZ rheology (see also [18]), including the transition from a viscous–plastic (consolidated ice) behaviour to the granular (MIZ) behaviour, e.g. following a wave-induced breakup event. He suggests discrete element modelling as a powerful tool for deriving emergent rheological properties of the MIZ. Further, he highlights the transfer of radiation stress (momentum) from waves to the ice cover as a crucial and understudied component of MIZ dynamics, which is likely to play a role in generating dynamic instabilities and thickening the ice cover through compression (see also [19]). He explains how the transfer of radiation stress may have wide-scale impacts on the atmosphere–ice–ocean system, such as compacting and thickening an initially thin and diffuse ice edge, and promotes the cascading effects this type of process may have as a key research question.

Thomson [15] takes the wave perspective on energy and momentum transfers in the MIZ, through the analogy of the MIZ to a surf zone, and in the context of the trend for increased Arctic wave climates. He describes two processes for future research focus in unravelling the role of MIZ dynamics in the atmosphere–ice–ocean feedback system: the transfer wave radiation stress to the ocean (as well as the ice cover), which can drive ocean mixing; and the transfer of wave energy to the ice and ocean, which can drive upper-ocean turbulence, and motivated by preliminary findings gained from *in situ* observations. He uses a simple model to show the energy transfer is significant for greater distances into the MIZ than the momentum transfer. He also highlights that the trend towards MIZ conditions in the Arctic Ocean is allowing more wave energy to reach the coastline and play a role in coastal erosion, so that future research should consider MIZ dynamics in an atmosphere–ice–ocean–land feedback system.

## 3. Pathways to influence climate assessments

The theme issue is conveniently timed to coincide with the final stage of the International Panel on Climate Change (IPCC) sixth assessment report (AR6). The IPCC provides the most authoritative scientific information on climate change for influencing high-level environmental and societal policies. In terms of MIZ science, the headline findings in the *Summary for Policymakers* from the AR6 Working Group I—The Physical Science Basis [20] and the earlier *Special Report on the Ocean and Cryosphere in a Changing Climate* [21] are on the responses of the Arctic and Antarctic sea ice covers to climate change. There is very high confidence that Arctic sea ice has decreased in extent (or area), thinned and become younger (i.e. it is becoming more MIZ-like), whereas Antarctic sea ice extents show no significant trend due to contrasting regional signals and interannual variability. Moreover, there is medium confidence that extreme wave heights have increased, exacerbated in the Arctic Ocean by sea ice loss. Future projections, based on Earth system models from the coupled model intercomparison project (CMIP5 [22] and CMIP6 [23]), focus on constraining estimates of when the Arctic will experience periods of complete ice loss, and the low confidence in Antarctic sea ice trends.

The sea ice and climate communities are showing increasing recognition of the MIZ as a crucial component of the ice-covered ocean [24,25], and that it is likely to play a key role in the responses of Arctic and Antarctic ice covers to climate change [26]. However, the MIZ is yet to have a substantial influence on the IPCC’s findings. The relevant sections of the full reports (in ch. 3 of [21] and ch. 9 of [20]) contain only isolated indirect references to MIZ processes. There is an indication of the positive feedback (discussed by Horvat [16]) in a comment that the thinning of Arctic sea ice leaves it more vulnerable to the increasing swell conditions [27], which contributes to further ice extent reductions. Two MIZ-focused studies [28,29] are cited when correlating regional trends in Antarctic ice coverage to winds, indicating the importance of MIZ dynamics on ice extents. The statement that *the importance of changing wave activity on sea ice is unclear due to limited process understanding* [30–32] is the most direct reference to the MIZ.

The last comment should be heeded by the MIZ community, as it expresses the opinion that there is not yet sufficient confidence in understanding of the MIZ and its trends for it to influence policy. In fact, there are very few studies that connect the MIZ to climate-scale behaviours of either the Arctic or Antarctic ice covers, with the most notable exception being by Kohout *et al.*’s [30] correlation of trends in the location of the Antarctic ice edge and trends in local wave energy. Integration of MIZ dynamics in Earth system models is the most accessible path to impact climate studies. It has been described as the *holy grail for MIZ research* by Squire [33], and there is considerable momentum towards the goal, as is evident from articles in the theme issue [9,11]. However, MIZ model components will potentially incur a considerable computational cost, including the need to couple wave and sea ice models. To ensure uptake by climate modellers, the MIZ community will have to provide evidence of the importance of the MIZ to model predictions. The evidence may be direct, e.g. showing the MIZ model has a major effect on ice predictions, as in Bennetts *et al.* [31], who showed 10–20% reductions in Antarctic sea ice volume during summer in a standalone sea ice model when wave-induce ice breakup is included, or indirect, e.g. as in Horvat [34], who used CMIP6 model outputs to show that the Arctic MIZ fraction is more weakly correlated to global mean temperature changes than ice extent, and thus a better indicator of sea ice model skill.

## 4. Concluding remarks

Squire [35] described the current phase of MIZ research as a resurgence. In truth, it has been far greater than that. Contemporary MIZ research is a coordinated, multidisciplinary, international research effort, which builds on the foundational understanding established by pioneering research efforts in the 1970s–1980s, and is motivated by the challenges and complexities created by climate change. The progress made over the past decade puts the field of MIZ dynamics in a position to push farther down the path of demonstrating its importance in the climate system, while tackling the fundamental research questions that remain [33].

## Data Availability

This article has no additional data.
